# Effects of menstrual cycle on hemodynamic and autonomic responses to central hypovolemia

**DOI:** 10.3389/fcvm.2024.1290703

**Published:** 2024-02-01

**Authors:** Vishwajeet Shankhwar, Janez Urevc, Bianca Steuber, Karin Schmid Zalaudek, Adam Saloň, Anna Hawliczek, Andrej Bergauer, Khawla Aljasmi, Asrar Abdi, Asmaa Naser, Maya Himeidi, Hanan Alsuwaidi, Stefan Du Plessis, Alawi Alsheikh-Ali, Catherine Kellett, Riad Bayoumi, Andrew Phillip Blaber, Nandu Goswami

**Affiliations:** ^1^ College of Medicine, Mohammed Bin Rashid University of Medicine and Health Sciences, Dubai, United Arab Emirates; ^2^ Faculty of Mechanical Engineering, University of Ljubljana, Ljubljana, Slovenia; ^3^ Division of Physiology, Otto Löwi Research Center of Vascular Biology, Immunity and Inflammation, Medical University of Graz, Graz, Austria; ^4^ Faculty of Health and Social Sciences, Inland Norway University of Applied Sciences, Lillehammer, Norway; ^5^ Department of Surgery, General Hospital (LKH) Südsteiermark, Wagna, Austria; ^6^ Department of Biomedical Physiology and Kinesiology, Simon Fraser University, Burnaby, BC, Canada; ^7^ Department of Integrative Medicine, Alma Mater Europea, Maribor, Slovenia

**Keywords:** follicular, luteal, estrogen, progesterone, sex-steroid hormones, heart rate variability, lower body negative pressure

## Abstract

**Background:**

Estrogen and progesterone levels undergo changes throughout the menstrual cycle. Existing literature regarding the effect of menstrual phases on cardiovascular and autonomic regulation during central hypovolemia is contradictory.

**Aims and study:**

This study aims to explore the influence of menstrual phases on cardiovascular and autonomic responses in both resting and during the central hypovolemia induced by lower body negative pressure (LBNP). This is a companion paper, in which data across the menstrual phases from healthy young females, whose results are reported in Shankwar et al. (2023), were further analysed.

**Methods:**

The study protocol consisted of three phases: (1) 30 min of supine rest; (2) 16 min of four LBNP levels; and (3) 5 min of supine recovery. Hemodynamic and autonomic responses (assessed via heart rate variability, HRV) were measured before-, during-, and after-LBNP application using Task Force Monitor® (CNSystems, Graz, Austria). Blood was also collected to measure estrogen and progesterone levels.

**Results:**

In this companion paper, we have exclusively assessed 14 females from the previous study (Shankwar et al., 2023): 8 in the follicular phase of the menstrual cycle (mean age 23.38 ± 3.58 years, height 166.00 ± 5.78 cm, weight 57.63 ± 5.39 kg and BMI of 20.92 ± 1.96 25 kg/m^2^) and 6 in the luteal phase (mean age 22.17 ± 1.33 years, height 169.83 ± 5.53 cm, weight 62.00 ± 7.54 kg and BMI of 21.45 ± 2.63 kg/m^2^). Baseline estrogen levels were significantly different from the follicular phase as compared to the luteal phase: (33.59 pg/ml, 108.02 pg/ml, respectively, *p* < 0.01). Resting hemodynamic variables showed no difference across the menstrual phases. However, females in the follicular phase showed significantly lower resting values of low-frequency (LF) band power (41.38 ± 11.75 n.u. and 58.47 ± 14.37 n.u., *p* = 0.01), but higher resting values of high frequency (HF) band power (58.62 ± 11.75 n.u. and 41.53 ± 14.37 n.u., *p* = 0.01), as compared to females in the luteal phase. During hypovolemia, the LF and HF band powers changed only in the follicular phase F(1, 7) = 77.34, *p* < 0.0001 and F(1, 7) = 520.06, *p* < 0.0001, respectively.

**Conclusions:**

The menstrual phase had an influence on resting autonomic variables, with higher sympathetic activity being observed during the luteal phase. Central hypovolemia leads to increased cardiovascular and autonomic responses, particularly during the luteal phase of the menstrual cycle, likely due to higher estrogen levels and increased sympathetic activity.

## 1 Introduction

Central hypovolemia, characterized by a reduction in blood volume within the central circulation (1), poses significant challenges to the cardiovascular system (2). The lower body negative pressure (LBNP) technique can be used to assess cardiovascular responses and stability during central hypovolemia (3, 4). The application of LBNP shifts the blood from the upper body to the lower body and leads to a decrease in the amount of blood returning to the heart (5, 6). The release of LBNP is rapidly followed by returning of blood from the lower limbs to the torso region in healthy young adults (7).

Hemodynamic and autonomic changes in response to LBNP have been extensively studied in both males and females (1, 3, 8). However, a few studies have reported the influence of menstrual phases on responses to central hypovolemia. Previous studies have indicated an increase in both progesterone and estrogen levels during the follicular and luteal phases (9–12), although some studies have reported contradictory results (13–15). Yazar et al., measured heart rate variability (HRV) in thirty women during their different phases of the menstrual cycle. The study concluded that markers of parasympathetic tone in HRV were not affected by the menstrual phase (16). Zambotti et al., reported the involvement of progesterone in cardiac autonomic control during sleep in women with and without premenstrual syndrome (17). Another study suggested there was no influence of menstrual phase during resistance exercise training (13), cold pressure test (11) and orthostatic intolerance (10). Yet, Antero and colleagues observed influence of the menstrual cycle on the training, performance, and overall well-being of elite rowers (9). Similar studies have been performed with inconsistent results reported regarding the role of menstrual phase on cardiovascular and autonomic responses (9–11, 14, 16–19). Furthermore, there is limited knowledge regarding how the menstrual phases affect the hemodynamic and autonomic parameters during application of LBNP.

This study aims to explore the influence of menstrual phases on cardiovascular and autonomic responses in both resting and during the central hypovolemia induced by lower body negative pressure (LBNP) (6, 7, 20–22). This is a companion paper, in which data across the menstrual phases from healthy young females, whose results are reported in Shankwar et al., were further analysed (23). While the Shankhwar et al. (23) article was based on the differences between male and female gender during hypovolemia, the current paper examines the effects of menstrual phase during hypovolemia. It was hypothesized that due to the differences in autonomic activity at rest in different menstrual phases (11, 14, 17), there will be a difference in the hemodynamic variables. Furthermore, during central hypovolemia, differences in hemodynamic and autonomic responses will be seen between the menstrual phases.

Understanding the effects of menstrual phases on cardiovascular and autonomic responses to central hypovolemia will provide insights into the underlying mechanisms, which may cause females to have a greater incidence of collapse/syncope/orthostatic intolerance in different phases (3, 24). The results of this study are crucial for optimizing cardiovascular stability in female astronauts, especially when they return to Earth from the microgravity environment of spaceflight.

## 2 Materials and methods

This randomized controlled study was carried out in 2022 at the Medical University of Graz, Austria. Ethical approval for the study was granted by the Ethics Committee at the Medical University of Graz, Austria (Reference: EK 25-551 ex 12/13). All data collection adhered to good clinical practices and was in accordance with the Declaration of Helsinki by the World Medical Association. Before participating in the study, all participants provided written consent.

### 2.1 Participants

In this study, we utilized the same group of participants who had previously been investigated in a larger study in which male and female participants were assessed for hemodynamic and autonomic responses for LBNP application. In our efforts to investigate the influence of the menstrual cycle on hemodynamic and autonomic responses to central hypovolemia, we employed the same cohort of participants previously examined in the initial published study. The recruitment inclusion and exclusion criteria are detailed in Shankwar et al. (23). Briefly, this study recruited young female participants, specifically targeting individuals aged between 18 and 30 years. Each participant was asked about their menstrual phase which was classified into the following two categories: 3–11 days after the onset of menses (follicular phase) and 21–27 days after the onset of menses (luteal phase) (15, 25).

### 2.2 Protocol

The detailed protocol and laboratory conditions are available in (23). Briefly, the study consisted of three phases: a 30 min rest period (baseline), followed by a 16 min session involving various levels of LBNP, and finally a 5 min recovery phase. An intravenous cannula was placed in an antecubital vein for blood sampling 30 min prior to recording of resting hemodynamic and autonomic variables. Once collected, the blood samples were sent to the testing laboratory to measure estrogen and progesterone using commercially available ELISA kits.

### 2.3 Data acquisition device

Continuous monitoring of physiological signals, including blood pressure, electrocardiogram (ECG), and thoracic impedance measurements, were conducted using a Task Force® Monitor (TFM) 3040i manufactured by CNSsystems in Graz, Austria. For details, please see Shankwar et al. (23).

### 2.4 Data analysis

The data analysis is similar as mentioned in the previously published article (23). In brief, data from each participant was analysed at baseline, during the application of LBNP, and the recovery phase. The study focused on specific time intervals (selected as 1–6), each lasting 10 s. These intervals included the last 10 s of supine rest (baseline), each LBNP levels, and recovery. The collected signals underwent processing to extract the hemodynamic and autonomic parameters such as heart rate, systolic blood pressure, diastolic blood pressure, mean arterial pressure, stroke index, cardiac index, total peripheral resistance index, as well as LF-RRI (R-R intervals) and HF-RRI band power derived from heart rate variability signals (26–29).

### 2.5 Statistical analysis

The statistical analysis of data obtained from participants in the follicular and luteal phases was performed by employing OriginPro Lab 2022 software. To assess normality in the hemodynamic and autonomic data, the Kolmogorov–Smirnov test was utilized. Statistical variations at baseline, different levels of LBNP, and during recovery were evaluated through repeated measure ANOVA and Bonferroni tests (*p* < 0.05). To evaluate the significance between follicular aand luteal phase, all time point values were compared between the phases using the Student *t*-test (*p* < 0.05). Additionally, we compared baseline hemodynamic and autonomic variables and hormone levels between menstrual phases using the Student *t*-test (*p* < 0.05).

## 3 Results

**Figure 1:**
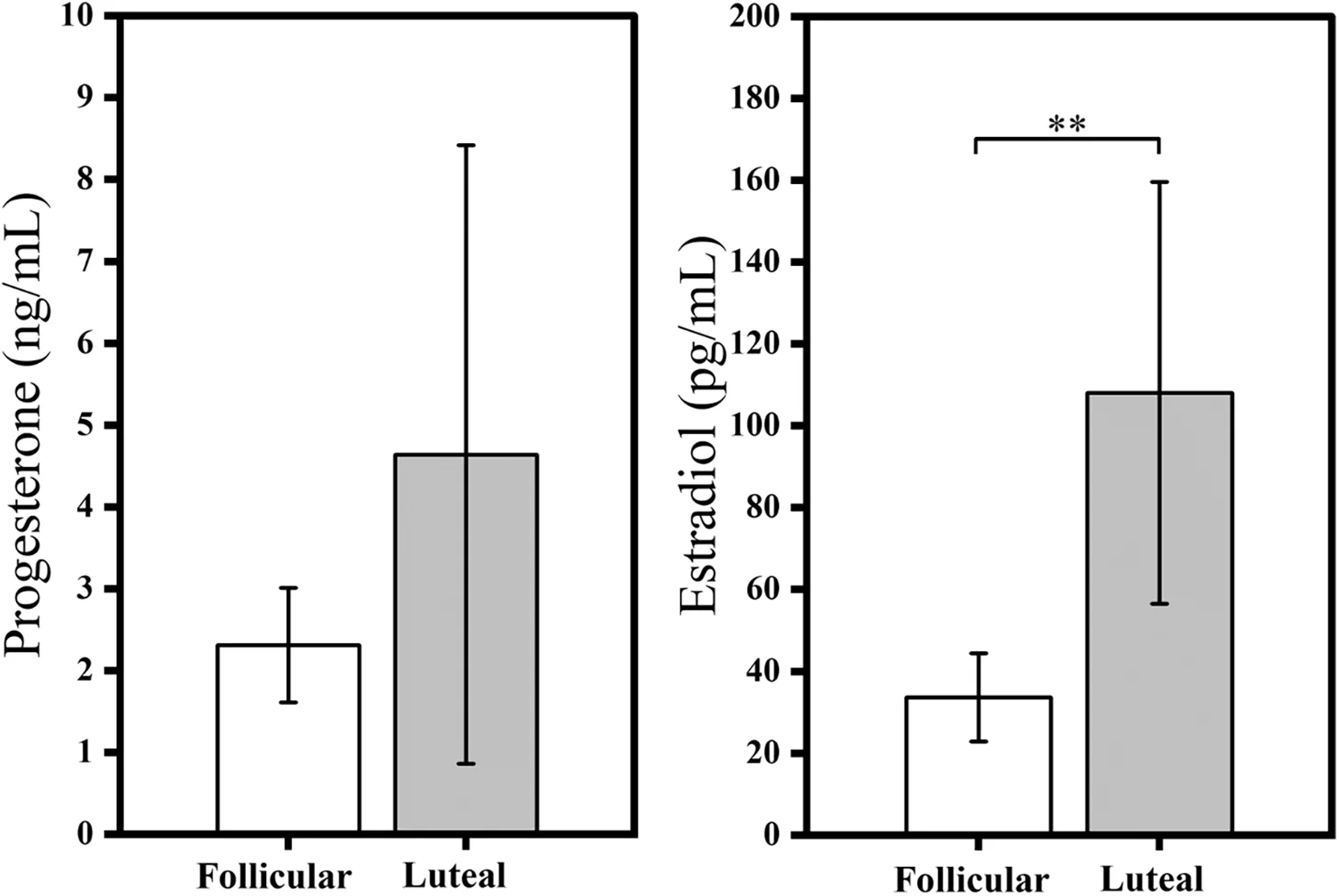
Comparisons of progesterone and estrogen (estradiol) hormones in the menstrual phases. **p < 0.01 significant differences between follicular and luteal phases.

A total of 21 female participants were enrolled in the study, from which 14 constituted the final analysis cohort (4 were excluded for not completing the protocol after developing fainting symptoms, and 3 were excluded due to the presence of noise from movement artifacts that precluded accurate recordings). Among the 14 participants in the analysis cohort, 8 were in the follicular phase (mean ± standard deviation values: age 23.38 ± 3.58 years, height of 166.00 ± 5.78 cm, weight of 57.63 ± 5.39 kg and BMI of 20.92 ± 1.96 kg/m^2^), and 6 were in the luteal phase (age 22.17 ± 1.33 years, height 169.83 ± 5.53 cm, weight 62.00 ± 7.54 kg and BMI of 21.45 ± 2.63 kg/m^2^). The hormone concentrations from both menstrual phases are shown in Figure 1. A significant increase in estrogen level from the follicular phase (33.59 pg/ml) to the luteal phase (108.02 pg/ml, *p* = 0.01) was observed. However, no significant alternation was observed in progesterone levels.

### 3.1 Effect of menstrual phases on resting hemodynamic and autonomic variables

**Figure 2:**
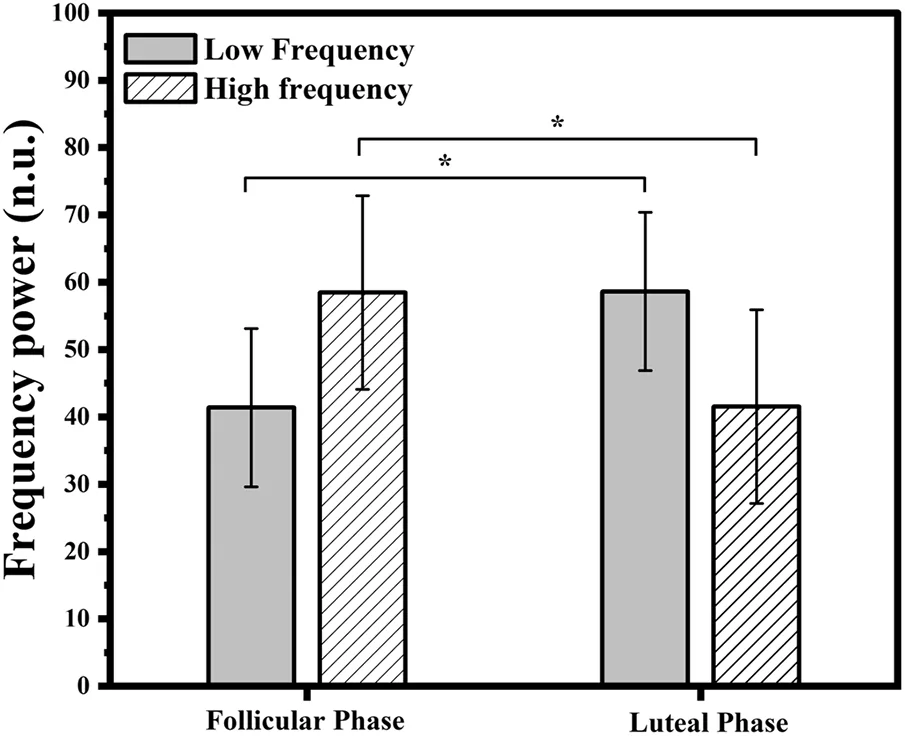
Resting autonomic variables (low and high frequency power) in follicular (*n* = 8) and luteal phases (*n* = 6). *p < 0.05 significant differences between follicular and luteal phases.

Participants in the follicular phase showed significantly lower resting values of low-frequency band power (*p* = 0.01), but higher resting values of high-frequency band power (*p* = 0.01) of heart rate variability as compared to females of the luteal phase (Figure 2, Table 1). No significant variations were observed in the resting hemodynamic variables.

**Table 1:** Hemodynamic and autonomic variables at rest, during LBNP application and recovery in follicular (*n* = 8) and luteal phases (*n* = 6).

	Baseline	−10 mmHg	−20 mmHg	−30 mmHg	−40 mmHg	Recovery
Heart rate (BPM)						
Follicular Phase	68.72 ± 13.46	69.15 ± 13.59	72.13 ± 14.20	73.90 ± 14.36	**78.39 ** **±** ** 16.53** ***	67.10 ± 13.28
Luteal phase	68.88 ± 9.28	67.98 ± 7.07	**75.64 ** **±** ** 8.75** *	**77.83 ** **±** ** 7.10** **	**81.67 ** **±** ** 7.47** ***	66.37 ± 7.25
Systolic blood pressure (mmHg)						
Follicular phase	107.74 ± 13.14	107.86 ± 11.71	106.35 ± 12.01	103.14 ± 8.62	108.75 ± 8.56	106.88 ± 9.68
Luteal phase	114.90 ± 7.85	112.61 ± 5.82	113.44 ± 5.99	113.10 ± 6.88	114.48 ± 5.62	110.88 ± 5.28
Diastolic blood pressure (mmHg)						
Follicular phase	68.26 ± 11.81	69.82 ± 10.79	71.54 ± 10.46	70.40 ± 9.84	73.87 ± 8.38	69.24 ± 8.94
Luteal phase	72.54 ± 8.64	72.66 ± 7.56	73.27 ± 5.50	74.64 ± 5.13	74.72 ± 6.32	69.51 ± 9.78
Mean arterial pressure (mmHg)						
Follicular phase	81.71 ± 9.33	83.21 ± 9.42	83.14 ± 10.08	84.04 ± 7.95	84.79 ± 6.75	82.73 ± 9.79
Luteal phase	90.17 ± 8.49	89.32 ± 7.51	89.91 ± 6.85	90.05 ± 6.22	89.12 ± 5.68	86.06 ± 5.14
Stroke index(ml/m^2^)						
Follicular phase	58.58 ± 13.23	56.23 ± 13.11	51.46 ± 12.18	46.08 ± 10.23	**40.58 ** **±** ** 7.92** ^***^	56.99 ± 12.25
Luteal phase	53.28 ± 10.20	50.00 ± 9.43	**44.20 ** **±** ** 11.10** ^**^	**38.06 ** **±** ** 7.58** ^***^	**37.24 ** **±** ** 9.34** ^***^	50.39 ± 10.89
Cardiac index (L/min/m^2^)						
Follicular phase	3.92 ± 0.72	3.79 ± 0.81	3.57 ± 0.46	**3.28 ** **±** ** 0.26** ^*^	**3.07 ** **±** ** 0.18** ^***^	3.73 ± 0.74
Luteal phase	3.61 ± 0.52	3.37 ± 0.50	3.28 ± 0.54	**2.94 ** **±** ** 0.51** ^***^	**3.00 ** **±** ** 0.64** ^***^	3.29 ± 0.43
TPR index (dyn*sec/cm^5^*m^2^)						
Follicular phase	1,721.48 ± 472.58	1,827.92 ± 514.50	1,910.97 ± 470.99	1,986.35 ± 289.99	**2,227.06 ** **±** ** 243.45** ^***^	1,826.44 ± 502.80
Luteal phase	1,970.54 ± 483.89	2,094.80 ± 479.05	2,169.69 ± 524.92	**2,443.26 ** **±** ** 633.00** ^*^	**2,482.03 ** **±** ** 864.36** ^*^	2,058.71 ± 273.95
Low frequency-RRI (n.u.)						
Follicular phase	41.38 ± 11.75	44.25 ± 17.84	50.34 ± 18.69	50.87 ± 16.72	**54.23 ** **±** ** 19.64** ^*^	38.54 ± 14.74
Luteal phase	**58.47 ** **±** ** 14.37** **^$^**	44.96 ± 5.82	62.03 ± 6.86	68.50 ± 15.77	75.54 ± 13.38	50.85 ± 13.44
High frequency-RRI (n.u.)						
Follicular phase	58.62 ± 11.75	55.75 ± 17.84	49.66 ± 18.69	49.13 ± 16.72	**45.77 ** **±** ** 19.64** ^*^	61.46 ± 14.74
Luteal phase	**41.53 ** **±** ** 14.37** **^$^**	55.04 ± 5.82	37.97 ± 6.86	31.50 ± 15.77	24.46 ± 13.38	49.15 ± 13.44

Data is displayed as mean ± SD.

Significant values have been marked as bold.

* *p* < 0.05 vs. baseline.

** *p* < 0.01 vs. baseline.

*** *p* < 0.001 vs. baseline.

^$^ *p* < 0.05 significant differences between resting (baseline) follicular and luteal phases.

### 3.2 Effect of menstrual phases on hemodynamic responses to central hypovolemia

**Figure 3:**
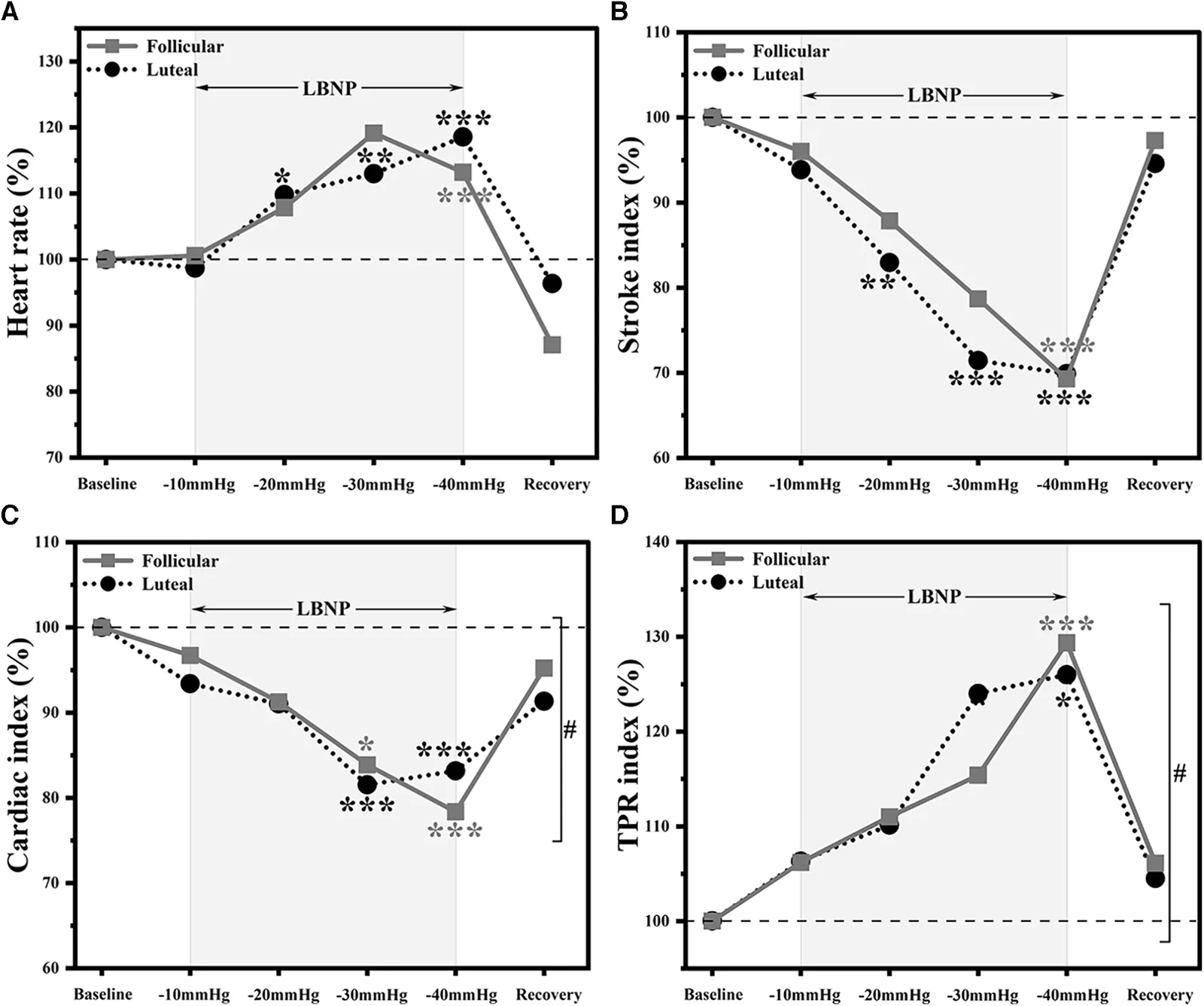
Hemodynamic responses to LBNP application in follicular (*n* = 8) and luteal phases (*n* = 6). Data normalised to baseline, (**A**) heart rate, (**B**) stroke index, (**C**) cardiac index and (**D**) total peripheral resistance (TPR) index. *p < 0.05 vs. baseline; **p < 0.01 vs. baseline; ***p < 0.001 vs. baseline. **^#^***p* < 0.05, overall significant differences between follicular and luteal phase participants responses to LBNP application (*p* < 0.05).

LBNP application significantly increased the heart rate in participants tested during the follicular phase F(1, 7) = 212.55, *p* < 0.0001 and luteal phase F(1, 5) = 603.64, *p* < 0.0001 (Table 1; Figure 3A). Arterial blood pressure values (systolic, diastolic, and mean arterial) were not significantly different after LBNP application compared to baseline. Stroke index was significantly decreased in follicular phase F(1, 7) = 177.46, *p* < 0.0001 and luteal phase F(1, 5) = 144.20, *p* < 0.0001 (Table 1, Figure 3B). Cardiac index was also significantly decreased in follicular phase F(1, 7) = 437.46, *p* < 0.0001 and luteal phase F(1, 5) = 264.12, *p* < 0.0001, (Table 1, Figure 3C). In contrast, total peripheral resistance index increased significantly during the follicular phase F(1, 7) = 108.04, *p* < 0.0001 and the luteal phase F(1, 5) = 196.26, *p* < 0.0001 (Table 1, Figure 3D). The influence of menstrual phases was observed in cardiac (*p* = 0.04) and total peripheral resistance (*p* = 0.01) indices during LBNP application (Table 1, Figures 3C,D). However, no effect was observed in heart rate, blood pressures and stroke index.

During the application of LBNP, we noted a significant increase in heart rate at −40mmHg relative to the baseline in both the follicular and luteal phases, with the increase being more pronounced in the luteal phase, as illustrated in Figure 3A. The stroke index experienced a reduction to 70% of the baseline value in both menstrual phases, and these values returned to the baseline level within five minutes of the recovery period. Notably, the decline in cardiac index was more prominent in the follicular phase when compared to the luteal phase. Furthermore, we observed a substantial 30% increase from the baseline value in the total peripheral resistance index in both menstrual phases.

### 3.3 Effect of menstrual phases on autonomic responses to central hypovolemia

**Figure 4:**
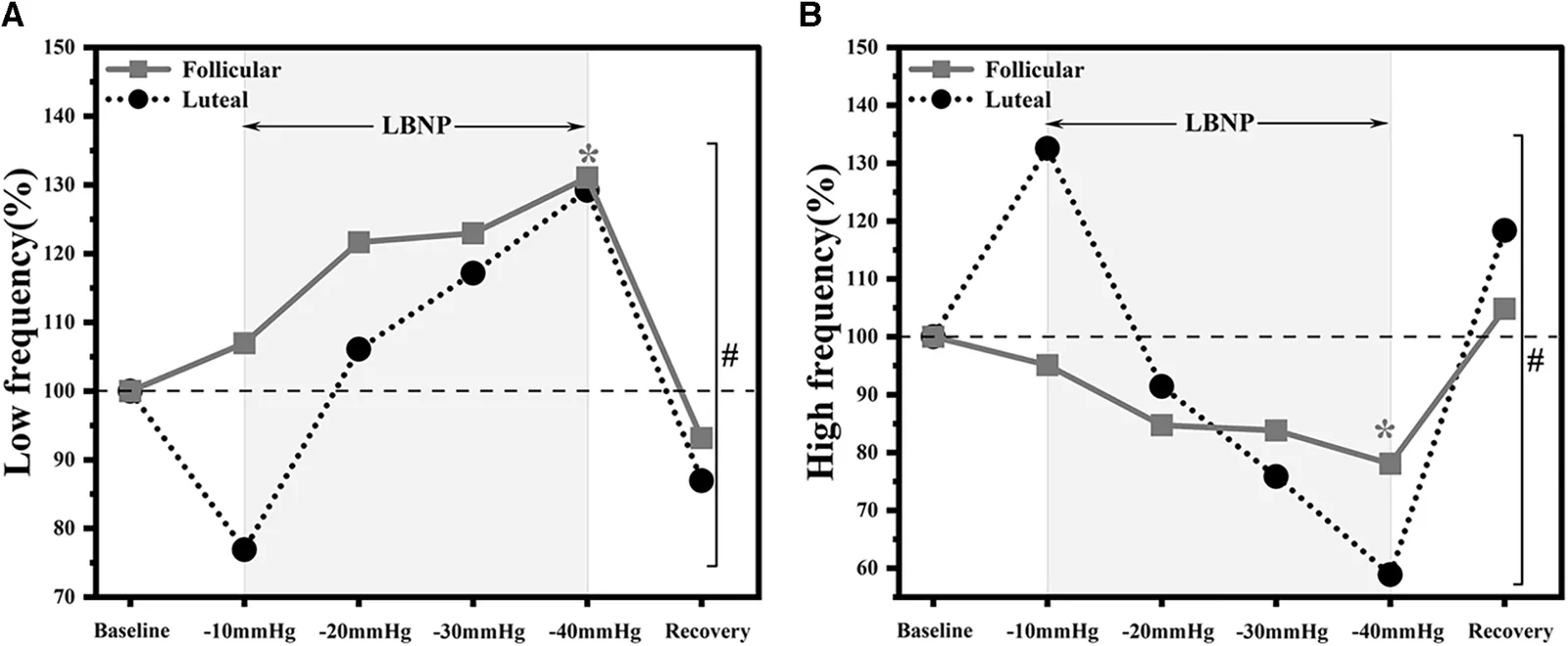
Autonomic responses to LBNP application in follicular (*n* = 8) and luteal phases (*n* = 6). Data normalised to baseline, (**A**) low-frequency and (**B**) high-frequency. *p < 0.05 vs. baseline. “#” overall significant differences between follicular and luteal phases participants responses to LBNP application (*p* < 0.05).

Low-frequency and high-frequency powers were significantly altered during LBNP only in the follicular phase F(1, 7) = 77.34, *p* < 0.0001 and F(1, 7) = 520.06, *p* < 0.0001, respectively (Table 1, Figure 4). The influence of menstrual phases on LF and HF responses during LBNP was found to be significant *p* = 0.01 and *p* = 0.01, respectively (Figure 4).

## 4 Discussion

This study is in the continuation of the previous published study that established the fundamental differences in hemodynamic and autonomic responses between males and females during central hypovolemia (23). The present study focuses on menstrual phase and hormonal fluctuations to better understand the physiological responses during central hypovolemia. This study aimed to investigate how menstrual phases influence the resting hemodynamic and autonomic parameters and their responses to central hypovolemia induced by LBNP. The main outcomes of this research are as follows: (i) Menstrual phases do not affect resting hemodynamic variables but the resting autonomic variables; and (ii) Menstrual phases influence central hypovolemia induced hemodynamic and autonomic responses.

### 4.1 Effect of menstrual phases on resting hemodynamic and autonomic variables

Progesterone and estrogen hormones are known to change during phases of the menstrual cycle. In the follicular phase, the concentrations of both hormones are low. However, as the follicular phase progresses, the levels of estrogen and progesterone begin to increase. Estrogen hormone is substantially more abundant in the luteal phase compared to the follicular phase (11, 15). The standard deviations of both hormones are higher in the luteal phase compared to the follicular phase. This indicates that there is greater variability or dispersion in hormone levels during the luteal phase. The present study also revealed that the increase in the concentrations of hormones is accompanied by a shift in autonomic nervous system activity, with LF and HF values swapping their dominance (Figure 2, Table 1). In the follicular phase, parasympathetic activity (higher HF) is prominent, while in the luteal phase, there seems to be a shift towards sympathetic activity (higher LF), which aligns with results from previously reported studies (14). This finding suggests that the steroidal hormones present during different menstrual phases impact autonomic parameters during supine rest. Yasar et al., observed enhanced sympathetic activity in the luteal phase (16). Zambotti et al., also found that the menstrual cycle influences autonomic regulation during sleep, leading to a change in heart rate and vagal activity specifically during the luteal phase.

In summary our findings highlight the role of estrogen in modulating autonomic function. During the luteal phase, characterized by elevated estrogen levels, we observed an increased sympathetic activity, as evidenced by higher heart rate, blood pressure, total peripheral resistance, and LF band power values. These observations align with the concept that estrogen promotes sympathetic dominance, leading to intense cardiovascular responses. Conversely, the follicular phase, characterized by lower estrogen levels, demonstrated greater parasympathetic activity, as indicated by higher HF band power.

As mentioned in the results section, four participants did not complete the entire study protocol due to the development of fainting symptoms. It is interesting to note that all of them were in the follicular phase. Similar results were observed by another research group, they found a higher incidence of positive syncope cases among participants in the follicular phase compared to the luteal phase during the head-up tilt test (30).

### 4.2 Effect of menstrual phases on hemodynamic and autonomic responses during central hypovolemia

At −10 mmHg LBNP, no significant changes in hemodynamic and autonomic activity were observed in participants from both phases (Figure 3). As expected during hypovolemia, heart rate increased during higher levels of LBNP (−20 mmHg to −40 mmHg). A decrease in stroke index was observed, reaching its lowest point at −40 mmHg in both phases, without any noticeable phase-specific effects. Cardiac index decreased in both phases, but the points at which the lowest values were reached differed between the phases. In the follicular phase, the lowest point in cardiac index was reached at −40 mmHg, while in the luteal phase it was demonstrated at −30 mmHg. This indicates that sympathetic activity was higher in the luteal phase, aided the participants to tolerating the −40 mmHg LBNP level and counter the effects. Conversely, in the follicular phase, lower sympathetic activity resulted in a decreased counteraction, leading to a continuous decrease in cardiac index. A distinct pattern emerged in the total peripheral resistance index, with the maximum increment observed in the follicular phase. This may be attributed to the fact that high sympathetic activity counteracted the resistance increment in the luteal phase, suggesting a regulatory role of sympathetic activity in this response. This also reveals that the responses of cardiac and peripheral resistance indices during LBNP application were influenced by the menstrual phase (Figures 3C,D).

Our study's findings validated a greater increase in LF band power during LBNP in the follicular phase compared to the luteal phase. This observation can be attributed to the fact that females in the luteal phase already exhibited higher sympathetic tone at baseline, resulting in a smaller increase during LBNP application. Claydon et al. reported that blood pressure and heart rate responses during induced orthostatic hypovolemia (LBNP with head-up tilt) were not influenced by menstrual phases (10). It is interesting to notice that these findings align with the current study, even without a head-up tilt maneuver. Stickford et al. demonstrated that the sympathetic activity responses during head-up tilt were not influenced by the menstrual cycle (11), which contrasts with the findings of the present study. This discrepancy can be attributed to the differences in the protocols utilized and the inclusion of participants who were not classified as healthy. According to Antero et al., athletic females tend to underperform and have fewer top scores in sports when they have menstrual symptoms, which usually happen before and during their periods (9). The results of the present study support this finding, as it revealed that low sympathetic activity in the follicular phase led to poorer performance during LBNP. It was found that participants in the luteal phase exhibited higher sympathetic activity and better tolerance to LBNP than those in the follicular phase. It was also found that estrogen and progesterone hormones influenced the hemodynamic and autonomic responses during LBNP. The study confirmed previous findings that menstrual symptoms impair women's performance during LBNP. Overall, the observed increases in heart rate, blood pressure, and total peripheral resistance with higher levels of LBNP were consistent with the expected physiological response to increased vascular load. Especially, the luteal phase exhibited amplified cardiovascular reactivity to LBNP application, possibly due to the combined influence of elevated estrogen levels and a more sympathetic-dominant state.

We have previously reported that the sympathetic reactivity is lower in females as compared to males during central hypovolemia (23). In present study, the sympathetic reactivity was high in follicular phase females during central hypovolemia as resting sympathetic activity was relatively low as compared to follicular phase. Taken as a whole, future studies should enrol participants of both sexes. In studies where females are included, the menstrual phase of each participant should be documented and taken into consideration during data interpretation. Understanding the impact of hormone-driven autonomic variations holds significant clinical implications. The elevated cardiovascular responses during the luteal phase may contribute to the increased incidence of conditions such as hypertension and orthostatic intolerance reported in some females. Moreover, the observed patterns could have implications for stress-related disorders, as sympathetic overactivity is linked to conditions like anxiety and panic disorders. Understanding interventions based on menstrual cycle phases might be a novel approach to managing such conditions.

## 5 Conclusions and future directions

Menstrual phases have an influence on resting autonomic variables. Interestingly, the elevated autonomic responses to LBNP application were observed in follicular phase females. The reactivity to LBNP was lower in luteal phase, potentially attributed to the elevated sympathetic activity during that phase. These findings suggest that estrogen and progesterone, which vary across the phases, play pivotal roles in shaping cardiovascular reactivity to the caudal fluid shift induced by LBNP application. Therefore, in future studies in which females are included, the menstrual phase should be taken into consideration.

Future research should consider exploring the impact of oral contraceptives on both hemodynamic and autonomic functions during rest and central hypovolemia to provide a comprehensive understanding of these dynamics in females.

## 6 Limitations

One notable limitation is that this study is that it is hypothesis generating and relatively small sample size. Despite these constraints, the present study lays a foundation for further investigation of menstrual cycle impact on responses to central hypovolemia emphasizing the need for replication with larger participant groups.

## Data Availability

The raw data supporting the conclusions of this article will be made available by the authors, without undue reservation.
